# Association of triglyceride glucose index and triglyceride glucose-body mass index with sudden cardiac arrest in the general population

**DOI:** 10.1186/s12933-024-02275-2

**Published:** 2024-05-18

**Authors:** Shuijing Zhang, Wenbing Liu, Bin Xu, Shuguang Wang, Zhongyan Du, Wenke Cheng

**Affiliations:** 1 The Affiliated Rehabilitation Hospital (Zhejiang Rehabilitation Medical Center)Zhejiang Chinese Medical University, Hangzhou, 310053 China; 2 The Third School of Clinical Medicine (School of Rehabilitation Medicine), Zhejiang Chinese Medical University, Hangzhou, 310053 China; 3 The Third Affiliated Hospital, Zhejiang Chinese Medical University, Hangzhou, 310053 China; 4 Zhejiang Greentown Cardiovascular Hospital, Hangzhou, 310053 China; 5 School of Basic Medical Sciences, Zhejiang Chinese Medical University, Hangzhou, 310053 China; 6 Key Laboratory of Blood-Stasis-Toxin Syndrome of Zhejiang Province, Hangzhou, 310053 China; 7 Zhejiang Engineering Research Center for “Preventive Treatment” Smart Health of Traditional Chinese Medicine, Hangzhou, 310053 China; 8 Medical Faculty, University of Leipzig, Liebigstr 27, Leipzig, 04103 Germany

**Keywords:** Triglyceride glucose, Triglyceride glucose-body mass index, Insulin resistance, Sudden cardiac arrest, Cohort study

## Abstract

**Background:**

Insulin resistance (IR) significantly contributes to cardiovascular disease (CVD) development. Triglyceride glucose (TyG) index and triglyceride glucose-body mass index (TyG-BMI) are recognised as convenient proxies for IR. However, their relationship with sudden cardiac arrest (SCA) remains unclear.

**Methods:**

This prospective cohort analysis included 355,242 UK Biobank participants with available TyG index and TyG-BMI data and no history of CVD. Cox proportional risk models assessed the association between the TyG index, TyG-BMI and SCA risk. Additionally, Accelerated Failure Time (AFT) models were employed to investigate the timing of SCA onset. The impact of dynamic increases in TyG index and TyG-BMI levels on SCA risk was examined using restricted cubic spline.

**Results:**

Over a median follow-up period of 165.4 months (interquartile range 156.5–174 months), 1,622 cases of SCA were recorded. Multivariate Cox regression analysis revealed a 9% increase in SCA risk per standard deviation increase in TyG index (adjusted hazard ratio (aHR) = 1.09, 95% confidence interval (CI) 1.04–1.15) and an 14% increase per standard deviation increase in TyG-BMI (aHR 1.14, 95% CI 1.09–1.2). AFT models indicated earlier median times to SCA occurrence with increasing quintiles of TyG index and TyG-BMI compared to the lowest quintile (P for trend < 0.05). SCA risk was linearly (P = 0.54) and non-linearly (P = 0.007) correlated with gradual increases in TyG index and TyG-BMI levels, respectively. Sex-stratified analyses showed stronger associations in women.

**Conclusions:**

Higher TyG index and TyG-BMI levels are associated with an increased SCA risk and earlier onset, particularly in women.

**Supplementary Information:**

The online version contains supplementary material available at 10.1186/s12933-024-02275-2.

## Introduction

Cardiovascular disease (CVD) is the primary cause of non-communicable disease-related deaths globally, accounting for approximately 17.9 million fatalities annually [1]. Despite the well-recognised risk factors such as hypertension, dyslipidaemia, diabetes mellitus and obesity, emerging evidence suggests a significant CVD risk even among individuals without these traditional risk factors [2, 3]. This underscores the imperative of identifying novel risk factors to refine CVD prevention and management strategies. Sudden cardiac arrest (SCA) represents a fatal cardiovascular emergency characterised by an abrupt cessation of heart function without preceding symptoms, posing challenges in prediction [4]. SCA results from sudden disruptions in the heart’s electrical activity, leading to ineffective blood pumping and rapid loss of consciousness and pulse. Without immediate interventions like cardiopulmonary resuscitation and defibrillation, SCA is overwhelmingly fatal. Global survival rates for out-of-hospital SCA is around 8.8%, with a slightly higher rate of 13% for in-hospital occurrences, underlining its public health significance and the urgency for interventions [5, 6].

Insulin resistance (IR) not only plays a pivotal role in CVD development but also serves as a predictive marker for CVD occurrence across both general and diabetic populations [7]. It manifests as diminished responsiveness of the body to insulin. While gold-standard diagnostic tests for IR, such as euglycemic insulin clamps and intravenous glucose tolerance tests, offer high accuracy, their practical utility is hampered by invasiveness and cost considerations [8]. In this context, the proposal of non-insulin-based IR surrogates has greatly simplified the testing process, improving the ease of assessment and general applicability. A variety of IR surrogates are known, including the visceral adiposity index (VAI), lipid accumulation product (LAP), triglyceride-glucose index (TyG index), TyG-body mass index (TyG-BMI), triglycerides/high-density lipoprotein cholesterol (TG/HDL-C) ratio, and metabolic score for IR (METS-IR). These indices are strongly associated with adverse cardiovascular events [9–12]. However, there may be differences in distinguishing IR among these proxies. It has been shown that the TyG index is superior to the TG/HDL-C ratio and VAI in the early identification of individuals with IR [13]. The study by Ahn et al. showed that among TyG, LAP, and VAI, the TyG index performed better in discriminating pre-diabetic and diabetic states in the general population [14]. Similarly, the TyG index was more useful than the TG/HDL-C ratio and METS-IR in predicting type 2 diabetes in a normoglycemic population [15]. In addition, the TyG index was superior to the HOMA2-IR index in identifying IR and was closely associated with IR-related fat distribution, fat stores, metabolic parameters, and subclinical atherosclerotic markers [16]. Er LK et al. showed that among LAP, VAI, the TyG index, TyG-BMI, TyG-waist circumference, and adipokine levels and ratios, TyG-BMI was superior to other indices in the early IR identification [17]. TyG-BMI also outperformed other parameters in predicting IR when combined with obesity-related parameters such as BMI, waist circumference (WC), and waist-to-height ratio (WHtR) [18]. These findings suggest that the combination of TyG and BMI has a more pronounced advantage in the early identification of insulin resistance compared to other insulin resistance parameters. Therefore, in this study, we focused on the TyG index and TyG-BMI. TyG index emerges as a practical and accessible surrogate for IR assessment [7]. Moreover, given obesity’s prevalent association with both IR and CVD, amalgamating the TyG index with body mass index (BMI) to form the TyG-BMI index might afford a more precise IR evaluation and enhance cardiovascular risk prediction [19]. A substantial body of evidence has established significant associations between TyG, TyG-BMI and various cardiovascular conditions including atherosclerosis, coronary artery disease, heart failure, acute coronary syndrome and mortality [7, 20–23]. However, investigations on the association of these two indices with SCA incidence in the general population are lacking. Thus, this study aims to investigate the association of TyG and TyG-BMI with SCA to provide a comprehensive framework for SCA risk assessment and management and thereby advocate for personalised CVD prevention strategies.

## Methods

### Data source and study design

The UK Biobank initiative, a prospective cohort study spanning from 2010 to 2016 across the United Kingdom, served as the data source. The detailed methodology of the study has been previously documented [24]. Comprehensive baseline information encompassing demographic and clinical profiles, lifestyle and health details, medical history and biological specimens were collected through physical assessments, interviews and laboratory analyses. Ethical approval for the Biobank project was obtained from the North West Multi-Center Research Ethics Committee (REC reference: 11/NW/0382), and all participants provided informed consent. Further information regarding the study is available on the UK Biobank website (http://www.ukbiobank.ac.uk).

This prospective cohort study initially enrolled 428,876 participants with complete TyG and TyG-BMI data and no history of SCA. Subsequently, participants with pre-existing cancer (n = 40,850) or pregnancy (n = 111) at baseline were excluded. Additionally, individuals with a history of CVD (n = 31,530) were excluded to minimise confounding effects on SCA. To mitigate potential reverse causality, participants (n = 1143) with a follow-up duration of less than 2 years were also excluded. Consequently, a total of 355,242 individuals were included in the primary analysis. The study adhered to the principles outlined in the *Declaration of Helsinki*.

### Assessment of TyG index and TyG-BMI

Peripheral venous blood samples were collected from all participants at baseline following validated procedures by the UK Biobank study [25]. Blood sampling was randomised due to the diverse distribution of assessment centres and intended use for studying various diseases [25]. At the time of collection, the fasting duration and time since the last meal (number of hours) were recorded. Blood samples were analysed by the UK Biobank within 24 h of collection using standard haematological tests, with coefficients of variation less than 3% for triglycerides and less than 2% for glucose. The TyG index was computed as: ln [triglycerides (mg/dl) × glucose (mg/dl)/2]. BMI was calculated by dividing weight (kg) by height (meter) squared and the result was retained to two decimal places. The TyG-BMI index was calculated as TyG*BMI.

### Assessment of covariates

In the initial survey, participants self-reported information on age, sex, ethnic background, blood pressure, blood lipids, physical activity, Townsend Deprivation Index (TDI), chronic medical conditions, medication use and smoking and drinking habits. The TDI, which serves as an indicator of socio-economic status, integrates aspects like employment status, car and home ownership and household density. Notably, higher TDI scores reflect lower socio-economic status [26]. To assess diet-related risk factors, a cumulative dietary risk score was calculated using a similar approach as the previous UK Biobank study [27]. Specifically, nine food items were selected to calculate the diet score, namely processed meat, red meat, total fish, milk, spreads, cereal intake, table salt, water and fruit and vegetables. These food items were categorised into two groups based on adherence to the recommended standards (UK and European dietary guidelines). For each category of unhealthy eating habits, participants were awarded 1 point. On adding up the scores for each participant, a final dietary score ranging from 0 (healthiest) to 9 (unhealthiest) was delineated. The total metabolic equivalent (MET) minutes per week was derived from a modified International Physical Activity Questionnaire [28]. The menopausal status of women was determined based on the UKB touchscreen questionnaire. Additionally, BMI, blood pressure and lipid levels were determined following standardised procedures. Baseline comorbidities were determined based on self-reports during the initial questionnaire or verbal interviews at enrolment, hospital diagnoses, or procedural codes (Table S1).

### Assessment of outcomes

The UK Biobank has established algorithms for specific health outcomes by integrating data sources, including death registries, primary care records, hospital admissions and self-reports. SCA was identified based on diagnostic codes associated with hospital admissions and death registries. Initial and subsequent instances of SCA were determined by ‘first occurrences of health outcomes’, using the three-digit ICD-10 code I46 (field ID: 131,347). The observation period for each study participant extended from cohort enrolment until the onset of SCA, death, or censoring on 7th December 2022 (field ID: 131,346), whichever occurred first.

### Statistical analysis

Individuals’ baseline characteristics were stratified by TyG index and TyG-BMI quintiles for comparison. Missing categorical variables were treated as missing indicators, and continuous variables were imputed using the mean. Categorical variables were presented as frequencies and percentages, while continuous variables were expressed as mean and standard deviation (SD). Differences between quintiles were assessed using the chi-squared test for categorical variables and one-way ANOVA for continuous variables.

Throughout the observation period, the incidence of SCA across different TyG index and TyG-BMI quintiles was evaluated utilising Kaplan–Meier analysis and log-rank test. Cox proportional hazard models were employed to elucidate the relationship between the TyG index, TyG-BMI levels and SCA risk, deriving hazard ratios (HRs) and 95% confidence intervals (CIs). The Schoenfeld Residuals test confirmed adherence to the proportional hazard assumption. Confounders were identified using established a priori knowledge to infer causality [29, 30]. A directed acyclic graph (DAG) was constructed using the DAGitty online tool (http://www.dagitty.net) to identify confounders for adjustment in the models, resulting in a minimal sufficient adjustment set including sex, age, race, BMI, waist circumference, TDI, fasting duration, physical activity, diet score, diabetes, menopausal status, lowering lipids, antihypertensives, insulin, and drinking status (Fig. S1). The variables hypertension, blood pressure, smoking habits and lipids were considered potential mediator variables in the exposure-outcome association and were not adjusted for in the main analysis. To address the possibility of overfitting, the variance inflation factor (VIF) was used to quantify the extent of multicollinearity between the variables. There was significant multicollinearity between waist circumference and BMI (VIF > 10), so only BMI was adjusted for in the main analysis, as it is a widely used indicator of obesity. The multivariate Cox regression comprised three distinct models: Model 1, an unadjusted crude model; Model 2, adjusted for age, sex and race; and Model 3, an extension of Model 2 that further adjusted for a comprehensive set of variables including TDI, physical activity, fasting duration, diet score, smoking and drinking status, lipid-lowering drugs, insulin and diabetes. The importance of each variable in the full model was calculated using the partial chi-square statistic minus the predictor degrees of freedom. Furthermore, in this analytical framework, the TyG index and TyG-BMI were treated as categorical and continuous variables. In the categorical analyses, the lowest quintile was used as a reference to assess the change in risk of SCA as the quintile group increased, and the trend P value of the quintile as a continuous variable was also calculated. Additionally, when the TyG index and TyG-BMI were analysed as continuous variables, Z-score normalisation (mean = 0, SD = 1) was used to quantify the change in SCA risk associated with each increase in SD.

To further our understanding, we conducted subgroup analyses. Subgroup analyses were conducted based on age, race, TDI, fasting time, diabetes, medication use, smoking and drinking status to assess the effect of each SD increase in the TyG index and TyG-BMI levels on SCA risk.

Additionally, the Accelerated Failure Time (AFT) model was employed to investigate the potential impact of TyG index and TyG-BMI levels on the timing of SCA events. Unlike models based on the proportional hazard assumption, the AFT model provides insights into how covariates may accelerate or decelerate the event timeline. To better fit the right-skewed distribution of the data, a more adaptable Weibull distribution was employed (Figs. S1 and S2). In the context of the multivariate AFT model, using the lowest TyG index and TyG-BMI quintile (Q1) as the reference group, we assessed the effect of these incremental increases on the timing of SCA onset. The median difference in time to SCA onset between the two groups, quantified in months, was calculated by subtracting the comparison group from Q1. Negative values indicated a delay in SCA onset, whereas positive values indicated an earlier onset.

The investigation into the exposure-effect relationship between TyG index and TyG-BMI levels and SCA events was conducted using restricted cubic splines (RCS), with three strategically placed knots (10th, 50th and 90th percentiles). RCS facilitated a nuanced understanding of the relationship dynamics, with non-linearity assessed through the log-likelihood ratio test.

In the sensitivity analyses, several strategies were used to verify the robustness of the findings. (1) Multiple imputations of missing data were conducted using five sets of predictive mean matching and Markov chain Monte Carlo methods. (2) Participants with coronary artery disease were added to the study population. (3) Participants with less than 2 years of follow-up were excluded to reduce reverse causality. (4) To account for the important variable of menopause status in women, the analysis was stratified by sex and adjusted for menopausal status in women. (5) In multivariate Cox regression models, BMI was replaced with waist circumference to verify the robustness of the results Statistical analyses were conducted using the R software (version 4.2.0). We deemed a two-sided P value of less than 0.05 as indicative of statistical significance.

## Results

**Fig. 1:**
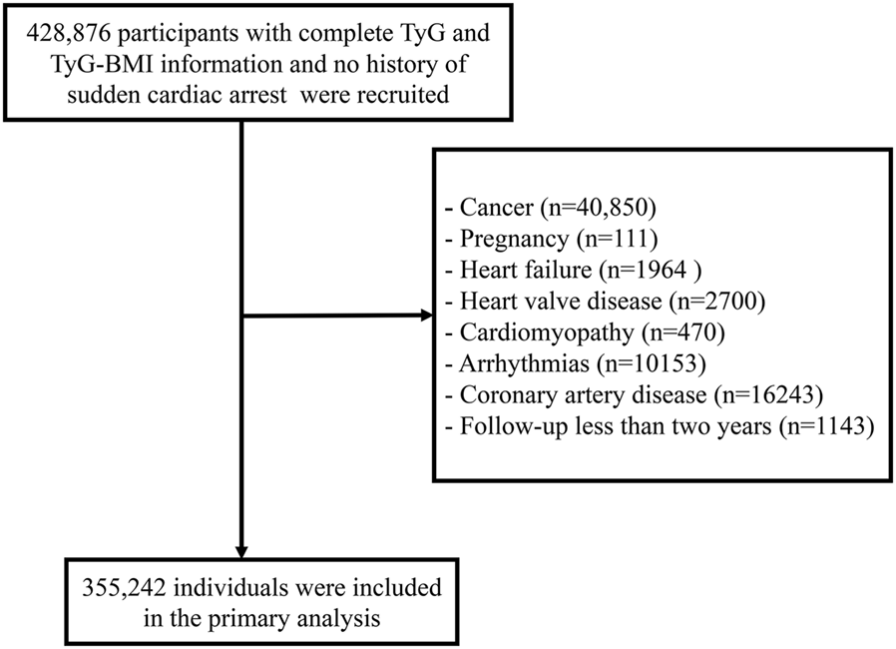
Flowchart of participant selection

The participant selection process is illustrated in Fig. 1, while Table S2 presents baseline characteristics of the 355,242 participants devoid of pre-existing SCA. Notably, physical activity data were missing for 22.32% of baseline records, with other variables showing missingness rates below 5% (Table S3). The mean age was 55.8 years (SD: 8.09), with 54.35% (193,060) being women and 93.89% (333,544) identifying as Caucasian. The average BMI stood at 27.32 kg/m^2^ (SD 4.72). Among the participants, a history of diabetes was reported by 4.3% (15,285), while 24.77% (88,009) had a history of hypertension. Additionally, 13.2% (46,822) were on cholesterol-lowering medications, and 0.87% (3084) reported insulin usage. Based on their initial TyG index and TyG-BMI levels, participants were categorised into quintiles. Significant differences across these groups were observed in several parameters, including age, sex, race, TDI, BMI, blood pressure, lipids levels, physical activity, history of chronic diseases and medications and smoking and drinking status (Tables 1 and 2).

**Table 1:** Stratified baseline characteristics according to TyG index quintile levels

TyG	Quintile 1 (≤ 8.2)	Quintile 2 (8.21–8.52)	Quintile 3 (8.53–8.81)	Quintile 4 (8.82–9.16)	Quintile 5 (≥ 9.17)	P-value
Number	69,347	72,653	71,136	70,838	71,268	
Age (years)	53.15 ± 8.14	55.61 ± 8.07	56.56 ± 7.95	56.96 ± 7.84	56.66 ± 7.83	< 0.001
TDI	− 1.33 ± 3.1	− 1.41 ± 3.03	− 1.39 ± 3.03	− 1.36 ± 3.05	− 1.17 ± 3.13	< 0.001
BMI (Kg/m^2^)	24.89 ± 3.9	26.29 ± 4.34	27.42 ± 4.55	28.39 ± 4.64	29.55 ± 4.68	< 0.001
Waist (cm)	81.50 ± 11.31	86.26 ± 12.15	90.15 ± 12.29	93.59 ± 12.16	97.85 ± 12.06	< 0.001
DBP (mmHg)	79.12 ± 9.9	81.43 ± 9.86	82.91 ± 9.91	84.02 ± 9.86	85.12 ± 9.81	< 0.001
SBP (mmHg)	131.07 ± 18.18	135.83 ± 18.35	138.57 ± 18.35	140.51 ± 17.93	142.48 ± 17.62	< 0.001
Physical activity (MET-min/week)	2813.06 ± 2403.2	2723.38 ± 2352.05	2669.55 ± 2340.48	2608.46 ± 2316.89	2500.67 ± 2315.66	< 0.001
TC (mmol/L)	5.33 ± 0.96	5.63 ± 1.01	5.79 ± 1.06	5.94 ± 1.12	6.08 ± 1.24	< 0.001
HDL-C (mmol/L)	1.70 ± 0.39	1.58 ± 0.37	1.46 ± 0.34	1.35 ± 0.31	1.20 ± 0.27	< 0.001
LDL-C (mmol/L)	3.19 ± 0.71	3.50 ± 0.76	3.67 ± 0.81	3.81 ± 0.86	3.85 ± 0.93	< 0.001
Fasting time (hours)	3.96 ± 2.79	3.91 ± 2.49	3.84 ± 2.4	3.74 ± 2.30	3.57 ± 2.23	< 0.001
Diet score	4.87 ± 1.54	4.97 ± 1.51	5.05 ± 1.5	5.15 ± 1.5	5.33 ± 1.5	< 0.001
Sex						< 0.001
Women	47,920 (69.1%)	44,764 (61.61%)	39,084 (54.94%)	34,227 (48.32%)	27,065 (37.98%)	
Men	21,427 (30.9%)	27,889 (38.39%)	32,052 (45.06%)	36,611 (51.68%)	44,203 (62.02%)	
Race						< 0.001
Non-Caucasian	5,448 (7.86%)	4,395 (6.05%)	3,918 (5.51%)	3,772 (5.32%)	4,165 (5.84%)	
Caucasian	63,899 (92.14%)	68,258 (93.95%)	67,218 (94.49%)	67,066 (94.68%)	67,103 (94.16%)	
Menopause status*						< 0.001
No	25,954 (54.16%)	18,495 (41.32%)	14,169 (36.25%)	11,533 (33.7%)	8,983 (33.19%)	
Yes	21,966 (45.84%)	26,269 (58.68%)	24,915 (63.75%)	22,694 (66.3%)	18,082 (66.81%)	
Hypertension						< 0.001
No	59,185 (85.35%)	57,900 (79.69%)	53,395 (75.06%)	50,310 (71.02%)	46,443 (65.17%)	
Yes	10,162 (14.65%)	14,753 (20.31%)	17,741 (24.94%)	20,528 (28.98%)	24,825 (34.83%)	
Diabetes						< 0.001
No	68,255 (98.43%)	71,263 (98.09%)	69,141 (97.20%)	67,745 (95.63%)	63,553 (89.17%)	
Yes	1,092 (1.57%)	1,390 (1.91%)	1,995 (2.80%)	3,093 (4.37%)	7,715 (10.83%)	
Smoker						< 0.001
Non-active	63,521 (91.68%)	65,767 (90.62%)	63,887 (89.89%)	62,910 (88.9%)	61,792 (86.84%)	
Active	5,761 (8.32%)	6,808 (9.38%)	7188 (10.11%)	7,856 (11.1%)	9,364 (13.16%)	
Drinker						< 0.001
Non-active	4,847 (7%)	5,363 (7.39%)	5,421 (7.63%)	5,647 (7.98%)	6,326 (8.89%)	
Active	64,434 (93%)	67,211 (92.61%)	65,654 (92.37%)	65,119 (92.02%)	64,830 (91.11%)	
Antihypertensives						< 0.001
No	62,733 (90.56%)	62,615 (86.28%)	58,774 (82.7%)	56,306 (79.57%)	53,072 (74.59%)	
Yes	6,543 (9.44%)	9,958 (13.72%)	12,295 (17.3%)	14,459 (20.43%)	18,079 (25.41%)	
Lowering lipids						< 0.001
No	64,387 (92.94%)	65,329 (90.02%)	62,218 (87.55%)	60,017 (84.81%)	56,061 (78.79%)	
Yes	4,889 (7.06%)	7,244 (9.98%)	8,851 (12.45%)	10,748 (15.19%)	15,090 (21.21%)	
Insulin						< 0.001
No	68,841 (99.37%)	72,295 (99.62%)	70,700 (99.48%)	70,240 (99.26%)	69,674 (97.92%)	
Yes	435 (0.63%)	278 (0.38%)	369 (0.52%)	525 (0.74%)	1,477 (2.08%)	

*TDI* Townsend deprivation index, *BMI* body mass index, *TC* total cholesterol, *LDL-C* low-density lipoprotein cholesterol, *HDL-C* high-density lipoprotein, *MET* metabolic equivalent task, *SBP* systolic blood pressure, *DBP* diastolic blood pressure

*Indicates the woman’s menopausal status

**Table 2:** Stratified baseline characteristics according to TyG-BMI quintile levels

TyG-BMI	Quintile 1 (< 197.5)	Quintile 2 (197.5–< 220.98)	Quintile 3 (220.98–< 244.15)	Quintile 4 (244.15–< 275.17)	Quintile 5 (≥ 275.17)	P-value
Number	71,049	71,048	71,048	71,048	71,049	
Age (years)	53.99 ± 8.17	55.79 ± 8.11	56.48 ± 8.05	56.61 ± 7.97	56.14 ± 7.84	< 0.001
TDI	− 1.44 ± 3.02	− 1.56 ± 2.97	− 1.48 ± 3	− 1.31 ± 3.07	− 0.88 ± 3.23	< 0.001
BMI (Kg/m^2^)	21.99 ± 1.63	24.75 ± 1.28	26.72 ± 1.41	28.98 ± 1.68	34.16 ± 4.16	< 0.001
Waist (cm)	75.40 ± 7.4	83.54 ± 7.54	89.46 ± 7.66	95.27 ± 7.85	105.80 ± 10.75	< 0.001
DBP (mmHg)	77.46 ± 9.58	80.71 ± 9.5	82.89 ± 9.56	84.78 ± 9.52	86.81 ± 9.58	< 0.001
SBP (mmHg)	129.82 ± 18.28	135.75 ± 18.23	138.91 ± 17.93	141.18 ± 17.69	142.93 ± 17.5	< 0.001
Physical activity (MET-min/week)	2833.99 ± 2388.27	2782.69 ± 2385.49	2703.13 ± 2373.91	2615.01 ± 2354.16	2377.74 ± 2205.48	< 0.001
TC (mmol/L)	5.55 ± 1	5.75 ± 1.06	5.83 ± 1.11	5.87 ± 1.15	5.78 ± 1.21	< 0.001
HDL (mmol/L)	1.72 ± 0.39	1.56 ± 0.36	1.44 ± 0.34	1.33 ± 0.31	1.24 ± 0.29	< 0.001
LDL (mmol/L)	3.33 ± 0.75	3.58 ± 0.81	3.70 ± 0.85	3.75 ± 0.87	3.67 ± 0.9	< 0.001
Fasting time (hours)	3.76 ± 2.46	3.76 ± 2.36	3.80 ± 2.4	3.83 ± 2.47	3.86 ± 2.56	< 0.001
Diet score	4.83 ± 1.55	4.95 ± 1.52	5.07 ± 1.5	5.21 ± 1.49	5.31 ± 1.49	< 0.001
Sex						< 0.001
Women	51,312 (72.22%)	40,848 (57.49%)	34,278 (48.25%)	31,325 (44.09%)	35,297 (49.68%)	
Men	19,737 (27.78%)	30,200 (42.51%)	36,770 (51.75%)	39,723 (55.91%)	35,752 (50.32%)	
Race						< 0.001
Non-Caucasian	3,925 (5.52%)	4,175 (5.88%)	4,573 (6.44%)	4,687 (6.6%)	4,338 (6.11%)	
Caucasian	67,124 (94.48%)	66,873 (94.12%)	66,475 (93.56%)	66,361 (93.4%)	66,711 (93.89%)	
Menopause status*						< 0.001
No	24,553 (47.85%)	16,107 (39.43%)	12,639 (36.87%)	11,475 (36.63%)	14,360 (40.68%)	
Yes	26,759 (52.15%)	24,741 (60.57%)	21,639 (63.13%)	19,850 (63.37%)	20,937 (59.32%)	
Hypertension						< 0.001
No	63,223 (88.99%)	58,773 (82.72%)	54,501 (76.71%)	49,760 (70.04%)	40,976 (57.67%)	
Yes	7,826 (11.01%)	12,275 (17.28%)	16,547 (23.29%)	21,288 (29.96%)	30,073 (42.33%)	
Diabetes						< 0.001
No	70,329 (98.99%)	69,859 (98.33%)	69,095 (97.25%)	67,840 (95.48%)	62,834 (88.44%)	
Yes	720 (1.01%)	1,189 (1.67%)	1,953 (2.75%)	3,208 (4.52%)	8,215 (11.56%)	
Smoker						< 0.001
Non-active	63,357 (89.24%)	63,850 (89.94%)	63,595 (89.61%)	63,478 (89.46%)	63,597 (89.64%)	
Active	7,636 (10.76%)	7,141 (10.06%)	7,372 (10.39%)	7,478 (10.54%)	7,350 (10.36%)	
Drinker						< 0.001
Non-active	5,150 (7.25%)	4,773 (6.72%)	5,051 (7.12%)	5,527 (7.79%)	7,103 (10.01%)	
Active	65,843 (92.75%)	66,218 (93.28%)	65,915 (92.88%)	65,428 (92.21%)	63,844 (89.99%)	
Antihypertensives						< 0.001
No	66,190 (93.24%)	62,935 (88.66%)	59,813 (84.29%)	56,018 (78.95%)	48,544 (68.43%)	
Yes	4,799 (6.76%)	8,052 (11.34%)	11,151 (15.71%)	14,933 (21.05%)	22,399 (31.57%)	
Lowering lipids						< 0.001
No	67,374 (94.91%)	64,654 (91.08%)	61,893 (87.22%)	59,244 (83.50%)	54,847 (77.31%)	
Yes	3,615 (5.09%)	6,333 (8.92%)	9,071 (12.78%)	11,707 (16.50%)	16,096 (22.69%)	
Insulin						< 0.001
No	70,647 (99.52%)	70,611 (99.47%)	70,530 (99.39%)	70,380 (99.2%)	69,582 (98.08%)	
Yes	342 (0.48%)	376 (0.53%)	434 (0.61%)	571 (0.8%)	1,361 (1.92%)	

*TDI* Townsend deprivation index, *BMI* body mass index, *TC* total cholesterol, *LDL-C* low-density lipoprotein cholesterol, *HDL-C* high-density lipoprotein, *MET* metabolic equivalent task, *SBP* systolic blood pressure, *DBP* diastolic blood pressure

*Indicates the woman’s menopausal status

### TyG index, TyG-BMI and incident SCA

**Fig. 2:**
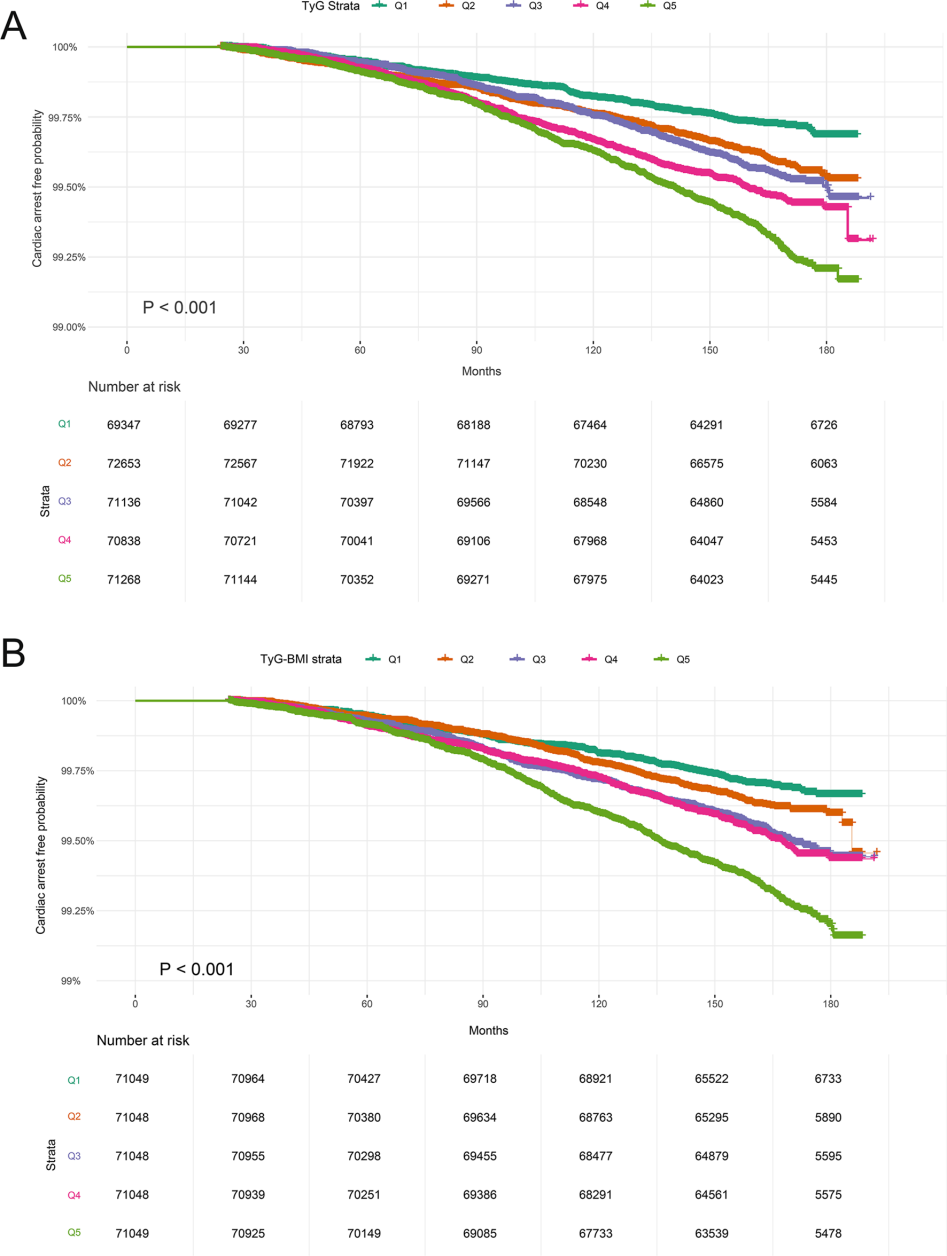
**A** Kaplan–Meier survival curves for SCA events in the TyG index quintile group. **B** Kaplan–Meier survival curves for SCA events in the TyG-BMI quintile group. Participants with less than 2 years of follow-up were excluded. *SCA* sudden cardiac arrest, *TyG index* triglyceride glucose index, *TyG-BMI* triglyceride glucose index–body mass index

**Fig. 3:**
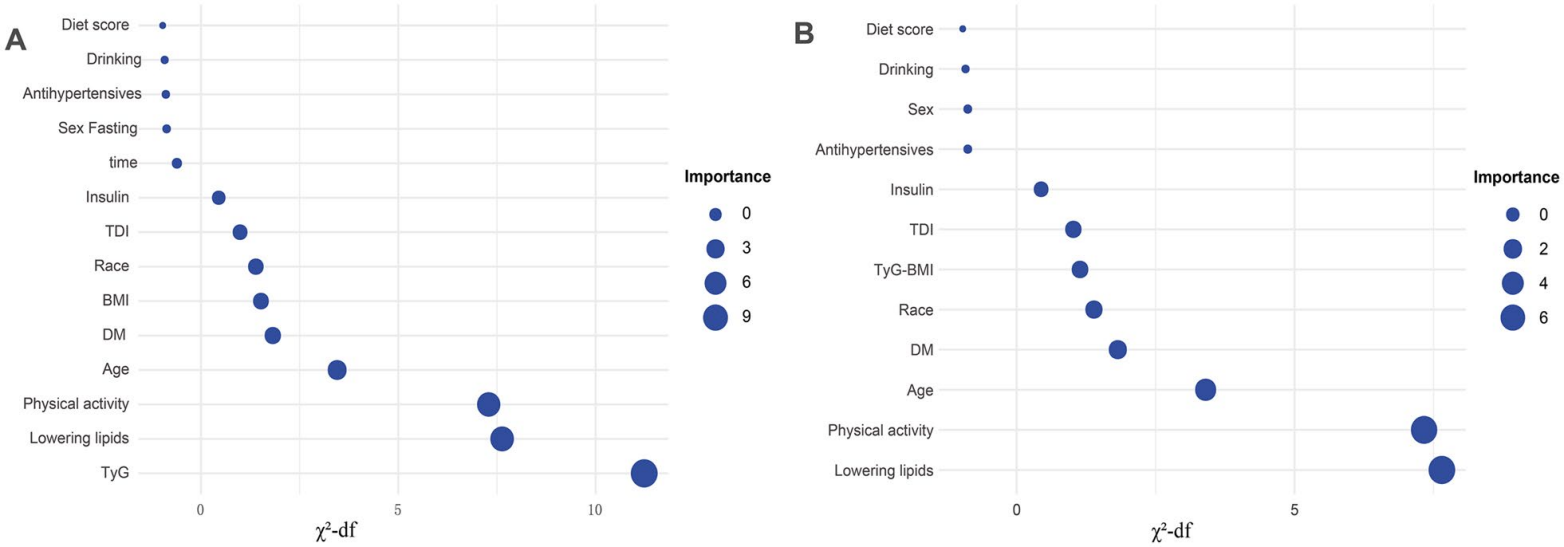
The relative importance of the variables in the model predicting the incidence of SCA, where importance is the chi-square statistic (χ^2^) minus the degrees of freedom (df) with respect to the predictor variable. *SCA* sudden cardiac arrest

**Fig. 4:**
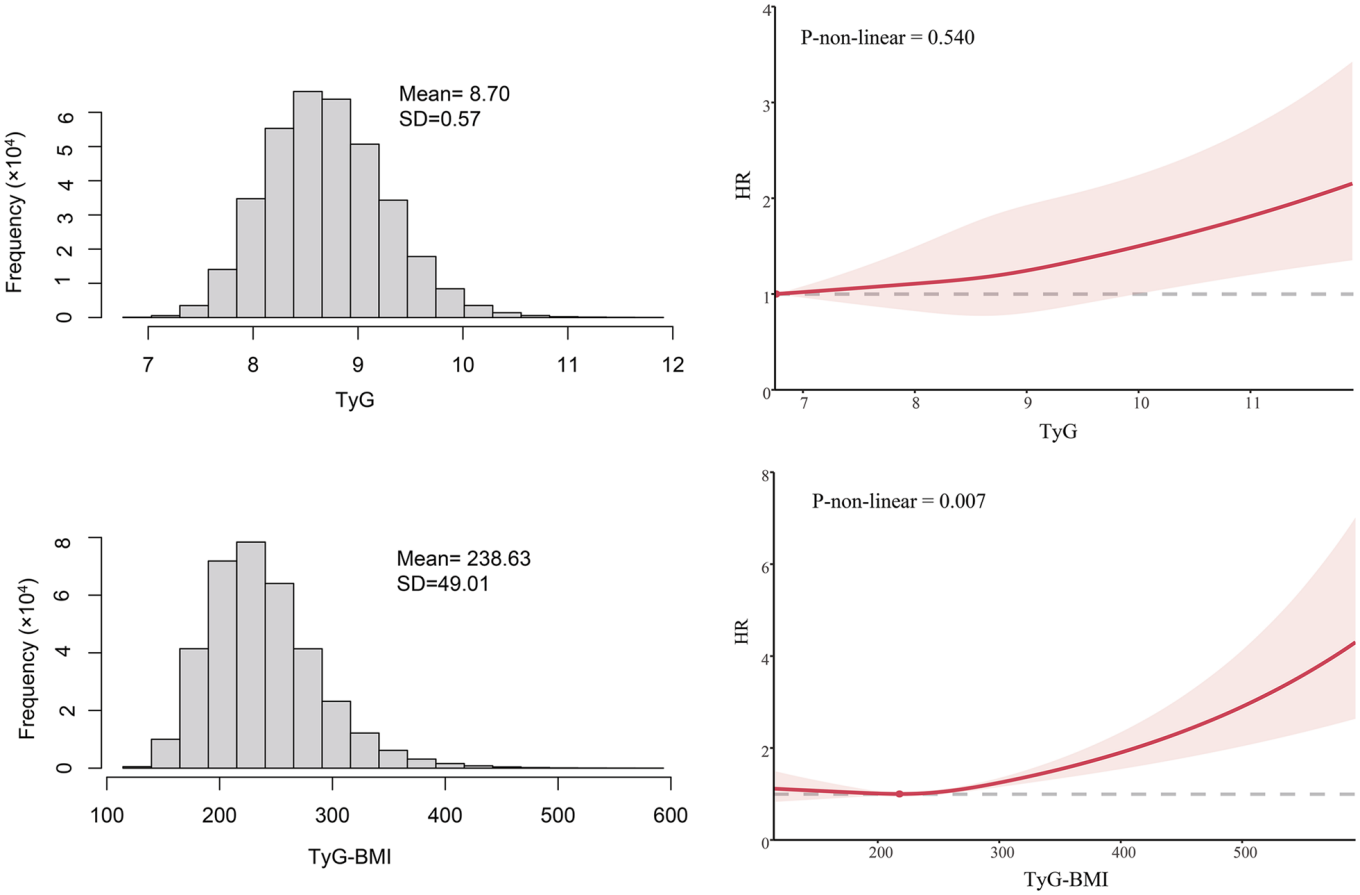
TyG index, TyG-BMI data distribution, and their restricted cubic spline with SCA risk. *SCA* sudden cardiac arrest, *TyG index* triglyceride glucose index, *TyG-BMI* triglyceride glucose index–body mass index

Over a median follow-up of 165.4 months (interquartile range 156.5–174 months; 4,806,994.5 person-years), 1,622 cases of SCA were documented. Kaplan–Meier analysis demonstrated a significant positive association with SCA risk as the TyG index and TyG-BMI quintiles increased (log-rank test P value < 0.001 for both; Fig. 2). Consistently, crude Cox regression analyses exhibited a consistent trend towards higher SCA risk with increasing TyG index and TyG-BMI quintiles in the unadjusted models (p for trend < 0.001; Table 3). The risk of SCA increased by 35% (HR 1.35; 95% CI 1.29–1.42) and 33% (HR 1.33; 95% CI 1.27–1.38) for each SD increase in TyG index and TyG-BMI, respectively. This association persisted after adjusting for age, sex and race. In fully adjusted models, the trend of increased SCA risk with higher TyG index and TyG-BMI quintiles continued (P for trend < 0.05), with each SD increase in TyG index and TyG-BMI increasing SCA risk by 9% (HR 1.09; 95% CI 1.04–1.15) and 14% (HR 1.14; 95% CI 1.09–1.2), respectively. Among the evaluated predictors, the TyG index emerged as the most crucial (χ^2^-df = 11.23; Fig. 3), while TyG-BMI exhibited moderate importance (χ^2^-df = 1.14; Fig. 3). Across the cohort, the mean values for the TyG index and TyG-BMI were 8.70 (SD: 0.57) and 238.63 (SD: 49.01), respectively. The RCS analysis revealed a linear association between the TyG index and SCA risk (P for nonlinear = 0.54; Fig. 4), contrasting with a nonlinear relationship between TyG-BMI and SCA risk (P for nonlinear = 0.007; Fig. 4).

**Table 3:** Multivariate COX regression analyses of TyG index and TyG-BMI with risk of SCA

	Model 1		Model 2		Model 3	
	HR (95% CI)	P value	HR (95% CI)	P-value	HR (95% CI)	P-value
TyG^a^						
Q1	Reference		Reference		Reference	
Q2	1.45 (1.21–1.75)	< 0.001	1.20 (1.0–1.44)	0.056	1.15 (0.96–1.38)	0.137
Q3	1.62 (1.35–1.94)	< 0.001	1.21 (1.01–1.47)	0.043	1.12 (0.93–1.34)	0.234
Q4	1.90 (1.60–2.27)	< 0.001	1.33 (1.12–1.59)	0.002	1.18 (0.98–1.41)	0.079
Q5	2.48 (2.10–2.94)	< 0.001	1.65 (1.39–1.96)	< 0.001	1.28 (1.07–1.53)	0.006
P for trend	< 0.001		< 0.001		0.007	
Per SD increase	1.35 (1.29–1.42)	< 0.001	1.21 (1.15–1.27)	< 0.001	1.09 (1.04–1.15)	< 0.001
TyG-BMI						
Q1	Reference		Reference		Reference	
Q2	1.23 (1.02–1.45)	0.028	0.96 (0.80–1.15)	0.685	0.95 (0.79–1.14)	0.563
Q3	1.56 (1.32–1.86)	0.003	1.10 (0.92–1.3)	0.302	1.04 (0.96–1.24)	0.676
Q4	1.62 (1.37–1.93)	< 0.001	1.10 (0.92–1.31)	0.27	0.97 (0.82–1.16)	0.743
Q5	2.28 (1.94–2.68)	< 0.001	1.68 (1.43–1.98)	< 0.001	1.25 (1.06–1.48)	0.01
P for trend	< 0.001		< 0.001		0.004	
Per SD increase	1.33 (1.27–1.38)	< 0.001	1.29 (1.23–1.35)	< 0.001	1.14 (1.09–1.2)	< 0.001

Model 1 has no variables adjusted

Model 2 adjusted age, sex, and race

Model 3 adjusted age, sex, race, TDI, physical activity, fasting time, diet score, diabetes, drinking status, lowering lipids drugs, insulin, and antihypertensives

*SCA* sudden cardiac arrest, *TyG index* triglyceride-glucose index, *TyG-BMI* triglyceride-glucose-body mass index, *SD* standard deviation

^a^Additional adjustment of body mass index in the model

### TyG index, TyG-BMI and time to SCA onset

**Fig. 5:**
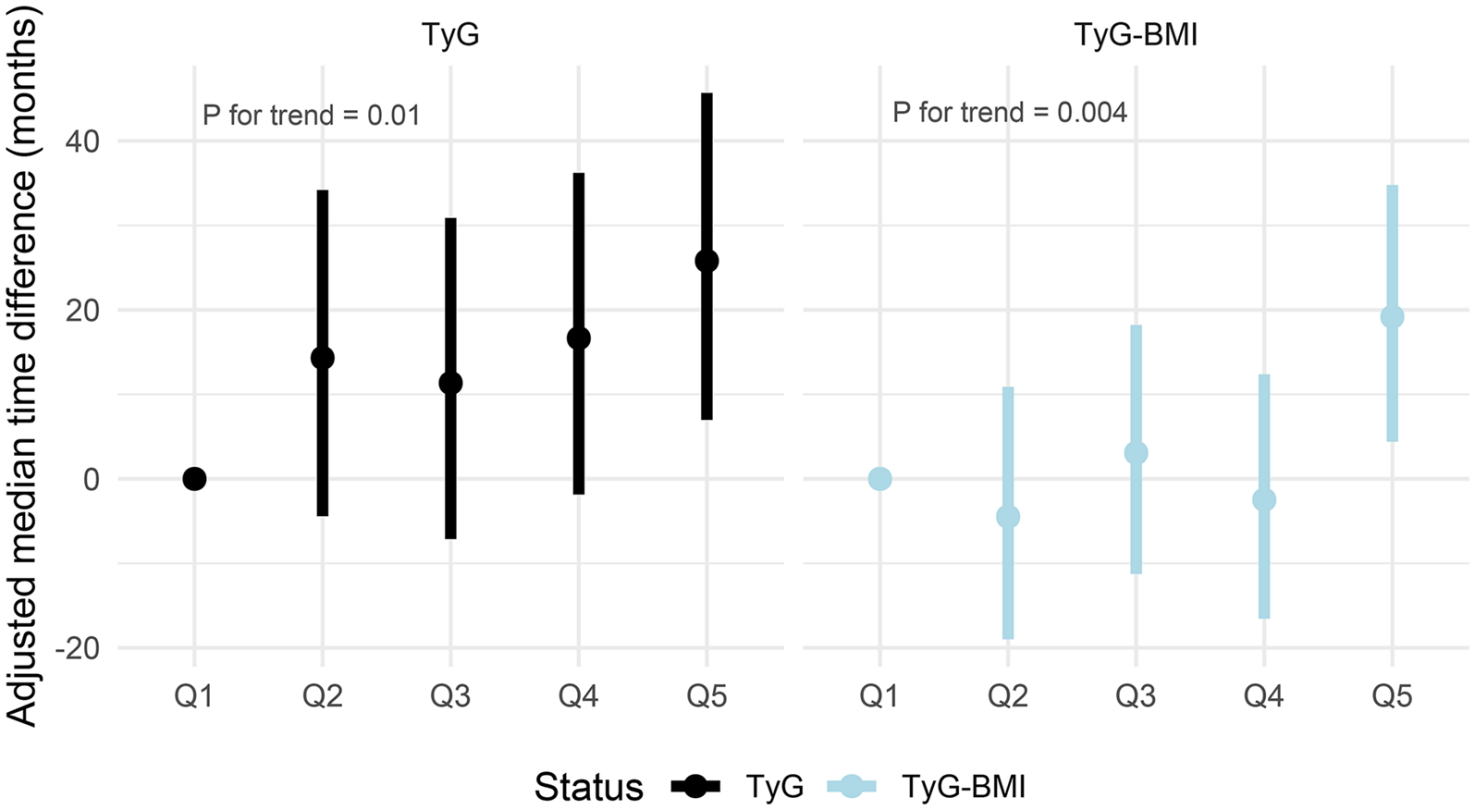
**A** Adjusted median difference in time to occurrence of SCA in the second, third, fourth, and fifth quintile groups compared to the quintile group with the lowest TyG index. **B** Adjusted median difference in time to occurrence of SCA in the second, third, fourth, and fifth quintile groups compared to the quintile group with the lowest TyG-BMI. Median difference = median occurrence time in reference group (Q1)—median occurrence time in comparison group. Negative values indicate a delay in the onset of events, while positive values indicate an earlier onset. In the AFT model, adjustments were made for age, sex, race, TDI, physical activity, fasting time, diet score, drinking status, lowering lipids drugs, antihypertensives, insulin, and diabetes. Additional adjustment of body mass index was made in the TyG index model. *TyG index* triglyceride glucose index, *TyG-BMI* triglyceride glucose index-body mass index

Analyses of the TyG index and TyG-BMI quintiles showed progressive advancement in time to SCA onset with increasing quintile levels (P for trend < 0.05; Fig. 5 and Table S4). Specifically, the median time to SCA onset was earlier in the second to fifth quintiles compared to the lowest TyG index quintile by 14.32 months, 11.34 months, 16.65 months and 25.8 months, respectively. A similar trend was observed for TyG-BMI, with a median time to SCA onset advanced in the second to fifth quintiles relative to the lowest quintile by − 4.48 months, 3.07 months, − 2.48 months, and 19.19 months, respectively.

### Subgroup analysis

**Fig. 6:**
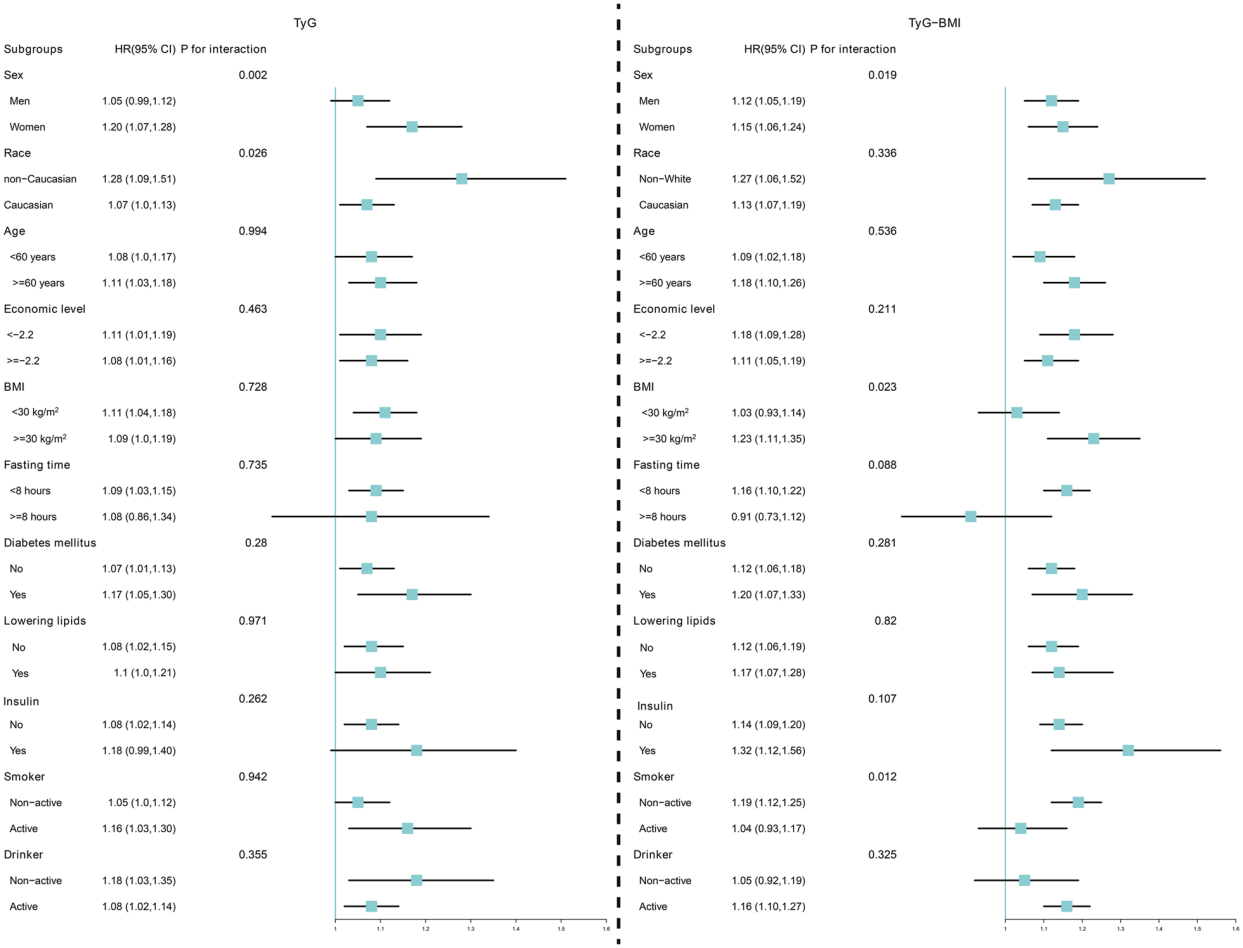
Changes in risk of SCA per 1 standard deviation increase in TyG index and TyG-BMI were assessed, stratified by different clinical characteristics. *SCA* sudden cardiac arrest, *TyG index* triglyceride glucose index, *TyG-BMI* triglyceride glucose index–body mass index

In subgroup analyses, positive associations between TyG index level and SCA risk remained consistent across most strata (P value for interaction > 0.05; Fig. 6), with significant interactions observed in the sex subgroup (P value for interaction = 0.002). Moreover, a mild interaction was also observed in the race group (P value for interaction = 0.026). Similarly, a positive association between TyG-BMI index and SCA risk was consistently present in most strata, but interactions were observed in the sex, BMI and smoking status subgroups (P value for interaction < 0.05). Sex showed an interaction between both the TyG index and TyG-BMI and SCA risk, with results suggesting a pronounced association in women. Therefore, we performed a sex-stratified RCS, which confirmed these findings, indicating higher SCA risk in women with dynamic increases in TyG index and TyG-BMI (Fig. S2). Moreover, AFT modelling revealed a more pronounced trend towards earlier SCA onset with rising TyG index and TyG-BMI quintiles in women compared to men (Table S5).

### Sensitivity analyses

In the sensitivity analyses, we included participants with coronary artery disease at baseline and those with less than 2 years of follow-up yielded consistent results (Table S6). Furthermore, the pooled results derived from the multiple imputations of five datasets were similar to the main findings (Table S7). Stratified analyses according to sex similarly showed a more pronounced risk of SCA in women than in men as TyG and TyG-BMI levels increased (Table S8). In addition, waist circumference replaced BMI in the multivariate Cox regression model, and the adjusted results were very similar to those of the main analysis (Table S9).

## Discussion

To the best of our knowledge, this study is the largest prospective cohort to date investigating the association between TyG index, TyG-BMI and SCA risk in the general population. Our findings reveal a significant correlation between elevated TyG index and TyG-BMI levels with an increased risk of SCA and earlier onset of SCA events. Notably, progressive increases in the TyG index and TyG-BMI were positively associated with SCA risk. These associations were more pronounced in women, underscoring a sex difference that suggests a stronger correlation in women than in men.

IR is a critical risk factor for T2DM, dyslipidemia, obesity and CVD [31]. The TyG index, known for its accessibility and accuracy in reflecting an individual’s IR status, has garnered attention in cardiovascular health research. Numerous studies have highlighted the link between IR and CVD progression, underscoring IR’s utility as a predictive marker for cardiovascular outcomes [32]. Notably, the TyG index has proven to be a reliable tool for assessing the progression of coronary artery calcification and determining the severity of coronary artery disease [33–35]. It is also positively correlated with the risk of heart failure, hypertension, atrial fibrillation and myocardial infarction [36–40]. Similarly, BMI serves as an indicator of both obesity and IR. A study from China reported a strong correlation between TyG-BMI and HOMA-IR in individuals without diabetes [17]. Similarly, a study from the Korean National Health and Nutrition Examination Survey compared the efficiency of four indicators—TyG index, TyG-BMI, TyG-WC, and TyG-WHtR—in reflecting IR. The results showed that as the level of TyG-WHtR increased, the odds ratio for the occurrence of IR was the highest. However, TyG-BMI was superior to the other parameters in predicting IR, with the largest area under the curve (AUC) of 0.748 among all subjects [18]. Moreover, TyG-BMI is also considered a marker of IR and has been shown to be associated with hypertension and hyperuricemia, which are closely related to CVD mortality [10, 41, 42].

SCA is a sudden, critical medical event that is challenging to predict. While several known predictors are associated with SCA risk, such as male sex, advanced age, diabetes and family history of coronary artery disease [43], these factors often lack sufficient predictive accuracy due to SCA’s low incidence rate [44]. SCA can be triggered by various heart-related or other health issues, with ventricular fibrillation and ventricular tachycardia being the most common heart-related causes [45]. In this study, to mitigate potential confounding effects of coronary artery disease on the results, participants with pre-existing coronary artery disease were excluded. In our regression models, both the TyG index and TyG-BMI showed a positive trend of association with increased risk of SCA, both as categorical and continuous variables. The RCSD analysis provides further visual evidence of this trend in risk dynamics. When assessing the importance of the TyG index and TyG-BMI in the model, the TyG index was identified as the most critical variable, with TyG-BMI regarded as relatively less important. This suggests that the TyG index contributes more significantly to SCA risk than TyG-BMI. Although BMI is associated with IR, it does not provide specific information on body fat distribution and therefore does not directly reflect the degree of IR. However, by combining abnormalities of glucose and lipid metabolism with BMI, TyG-BMI provides a more comprehensive risk assessment, suggesting that the combination of obesity and metabolic abnormalities has a greater impact on the increased risk of SCA. This explains the 9% increase in the risk of SCA per 1 SD increase in TyG index and the 18% increase in the risk of SCA per 1 SD increase in TyG-BMI. Moreover, sensitivity analyses included individuals with coronary artery disease and adjusted for coronary artery disease, revealing consistent results with the main analyses. These findings emphasise that the associations between the TyG index and TyG-BMI and SCA risk remain stable even after accounting for coronary artery disease, an important CVD factor.

IR and the incidence of SCA involve complex mechanisms. IR, a marker of metabolic dysfunction, exacerbates the risk of cardiovascular events [46]. Dyslipidemia, a consequence of IR, is characterised by elevated TG levels and reduced HDL cholesterol levels, both significant risk factors for CVD [47]. Hyperglycemia and hyperinsulinemia resulting from IR may further burden the cardiovascular system [48]. Concurrently, increased inflammation, commonly observed in IR, plays a pivotal role in atherosclerosis development, a core mechanism underlying sudden cardiac death [49]. IR contributes to CVD progression by increasing vascular stiffness and reducing NO bioavailability [32, 50]. Moreover, chronic IR induces notable changes in cardiac structure and function, including left ventricular hypertrophy and cardiac hypo-contractility, elevating the risk of cardiovascular morbidity and mortality [51]. Additionally, IR directly affects the heart’s electrophysiological properties, potentially increasing the risk of arrhythmias, precursors to sudden cardiac death [52]. A comprehensive understanding of these interrelationships is essential for formulating effective prevention and treatment strategies for CVD.

Sex is a crucial determinant in CVD development and progression, with growing attention on cardiovascular risk in women. Sex-specific analyses revealed a more pronounced association between IR and SCA in women, aligning with observations from previous studies indicating a stronger link between IR and adverse cardiovascular events in women [20, 37, 38]. Notably, consistent findings were observed across RCS and AFT models. Mechanistically, before menopause, women benefit from oestrogen’s protective effects, experiencing lower CVD risk compared to men. However, postmenopausal declines in oestrogen levels weaken this protection, increasing IR risk in women [53]. This shift in hormonal balance after menopause can affect lipid metabolism and insulin sensitivity, increasing the susceptibility to SCA in women [54]. Moreover, sex disparities in fat distribution, with women accumulating fat in hips and thighs while men favour abdominal deposition, contribute to age-related visceral fat accumulation in women, exacerbating IR and cardiovascular risk [55]. Inflammation and oxidative stress, key factors in IR and CVD, exhibit greater sensitivity in women, augmenting SCA risk [56]. Sex-related biases in CVD diagnosis and treatment may predispose women to mismanagement, escalating complication risks. Given IR’s central role in T2DM, studies indicate women tend to accumulate more cardiovascular risk before T2DM onset, amplifying SCA risk upon IR development [57].

### Strengths and limitations

This study boasts several strengths. It is a large-scale prospective design study with comprehensive long-term follow-up data, and causal identification of potential risk factors enhances the robustness of its findings. The utilisation of the AFT model to analyse the TyG index and TyG-BMI in terms of SCA onset time, along with validation through Cox regression, ensures result stability. However, several limitations warrant consideration. Being an observational cohort study, it cannot establish direct causal relationships. Despite adjustments for multiple potential risk factors, the influence of unobserved variables remains possible. Additionally, reliance on self-reported medical history introduces potential recall bias. Certain confounders, like oestrogen levels, were not accounted for due to data limitations. TyG index and TyG-BMI were calculated from baseline blood samples, with non-fasting blood glucose and triglycerides values being a significant limitation. Although fasting time was adjusted for, its impact on the results could not be eliminated. Nonetheless, stratified analyses indicated no significant interaction between fasting time of < 8 h and ≥ 8 h, implying that fasting time had limited influence on study outcomes. Our study population being from the United Kingdom necessitates further validation of result generalizability to other demographics.

## Conclusions

Elevated TyG index and TyG-BMI levels are linked to heightened SCA risk and earlier onset, with these associations particularly pronounced in women.

### Supplementary Information

Additional file1 (PDF 465 kb)

## Data Availability

Data can be accessed from a public and open repository. This study was conducted using the UK Biobank Resource, Application Number: 107335. Interested researchers can apply for access to the UK Biobank data at www.ukbiobank.ac.uk.
