# Supplementary material for: Association of triglyceride glucose index and triglyceride glucose-body mass index with sudden cardiac arrest in the general population
Source: Cardiovasc Diabetol. 2024 May 18;23:173. doi: 10.1186/s12933-024-02275-2 (PMC11102616; doi:10.1186/s12933-024-02275-2)

Supplement

Table S1. Diagnostic codes for diseases.
Table S2. Baseline characteristics of overall participants.
Table S3. Proportion of Missing Rates for Baseline Characteristics.
Table S4. Adjusted median time difference for SCA occurrence in the second, third, fourth, and highest quintile groups compared to the TyG index and TyG-BMI lowest quintile (Q1) groups.
Table S5. Adjusted median time difference for SCA occurrence in the second, third, fourth, and highest quintile groups compared to the TyG index and TyG-BMI lowest quintile (Q1) groups according to sex.
Table S6. Multivariate COX regression analyses of TyG index and TyG-BMI with risk of SCA.
Table S7. Multivariate Cox regression of per SD increase in TyG index and TyG-BMI and SCA risk after multiple imputations of five data sets.
Table S8. Multivariate Cox regression models stratified by sex to investigate the association of TyG and TyG-BMI levels with the incidence of SCA.
Table S9. Multivariate Cox regression model to study the relationship between TyG levels and SCA incidence.
Figure S1. Directed acyclic graph of the link between TyG index and TyG-BMI and the onset of SCA.
Figure S2. Restricted cubic splines of TyG index, TyG-BMI and risk of SCA in women and men.

Table S1. Diagnostic codes for diseases

Diseases	ICD-9 diagnosis	ICD-10 diagnosis	Self-report [a]	Primary Care [b]	Medication [c] / Operation code, self-reported [d]
Hypertension	401, 4010, 4011, 4019, 402, 4020, 4021, 4029, 403, 4030, 4031, 4039, 404, 4040, 4041, 4049, 405, 4050, 4051, 4059	I10-I13, I15	20002	Category 3000	Blood pressure medication / NA
Diabetes	2500, 25000, 25001, 25009, 2501, 25011, 25019, 2503, 2504, 2505, 25099	E10-E14	20002	Category 3000	Insulin/ NA
CAD	410, 4109, 411, 4119, 412, 4129, 413, 4139, 4140, 4148, 4149	I20-I25	20002	Category 3000	NA / Coronary angioplasty (PTCA) +/- stent; Coronary artery bypass grafts (CABG); Triple heart bypass
Heart failure	428, 4280, 4281, 4289	I50	20002	Category 3000	NA
cardiomyopathy	422, 4229, 425, 4251, 4254	I40-I43	20002	Category 3000	NA
Heart valve disease	394, 3940, 3942, 3949, 395, 3951, 3959, 396, 3969, 424, 4240, 4241, 4243, 4249	I05-I09, I34-I38	20002	Category 3000	NA / Aortic valve repair/replacement; Mitral valve repair/replacement; Other valve repair/replacement
Arrhythmia	426, 4260, 4261, 4263, 4264, 4265, 4266, 4267, 4269, 427, 4270, 4271, 4273, 4274, 4276, 4278, 4279	I44, I45, I47-I49	20002	Category 3000	NA / Pacemaker; Defibrillator insertion; Cardiac ablation

ICD: International Classification of Disease. NA, not applicable.

[a] 20002 is the data code used in UK Biobank: Non-cancer illness code, self-reported.

[b] This category contains data on primary care data recorded by health professionals working at general practices.

[c] Data-field 6153 and 6177.

[d] Data-Field 20004

Table S2. Baseline characteristics of overall participants (N=355,242).

Age (years)	55.80 ± 8.09
TDI	-1.33 ± 3.07
BMI (Kg/m^2)	27.32 ± 4.72
Waist (cm)	89.89 ± 13.27
DBP (mmHg)	82.53 ± 10.09
SBP (mmHg)	137.72 ± 18.51
Physical activity (MET-min/week)	2662.51 ± 2347.96
TC (mmol/L)	5.75 ± 1.11
HDL (mmol/L)	1.46 ± 0.38
LDL (mmol/L)	3.61 ± 0.85
Fasting time (hours)	3.80 ± 2.45
Diet score	5.07 ± 1.52
Sex	
Women	193,060 (54.35%)
Men	162,182 (45.65%)
Race	
Non- Caucasian	21,698 (6.11%)
Caucasian	333,544 (93.89%)
Hypertension	
No	267,233 (75.23%)
Yes	88,009 (24.77%)
DM	
No	339,957 (95.7%)
Yes	15,285 (4.3%)
Smoker	
Non-active	317,877 (89.58%)
Active	36,977 (10.42%)
Drinker	
Non-active	27,604 (7.78%)
Active	327,248 (92.22%)
Antihypertensives	
No	293,500 (82.71%)
Yes	61,334 (17.29%)
Lowering lipids	
No	308,012 (86.8%)
Yes	46,822 (13.2%)
Insulin	
No	351,750 (99.13%)
Yes	3,084 (0.87%)

Table S3. Proportion of Missing Rates for Baseline Characteristics.

Variables	Complete (Yes/No)	Missing rates (Number / %)
Age	No	
Sex	No	
Race	No	
TDI	Yes	441/ 0.12%
BMI	Yes	
Waist	Yes	724 / 0.2%
DBP	Yes	375 / 0.105%
SBP	Yes	376 / 0.106 %
Physical activity	Yes	79,315 / 22.32%
Menopause status	No	
TC	Yes	86 / 0.024%
HDL	Yes	82 / 0.023%
LDL	Yes	532 / 0.15%
Fasting time	No	
Diet score	Yes	13,136 / 3.7%
Sex	No	
Race	No	
Hypertension	No	
DM	No	
Smoker	No	
Drinker	No	
Antihypertensives	No	
Lowering lipids	No	
Insulin	No	

Table S4. Adjusted median time difference for SCA occurrence in the second, third, fourth, and highest quintile groups compared to the TyG index and TyG-BMI lowest quintile (Q1) groups.

Categories	Median Difference (months)	95 % Lower CI	95 % Upper CI
	**TyG[a]**		
Q2	14.32	34.19	-4.43
Q3	11.34	30.89	-7.12
Q4	16.65	36.23	-1.86
Q5	25.8	45.69	6.98
	**TyG-BMI**		
Q2	-4.48	10.89	-19
Q3	3.07	18.21	-11.26
Q4	-2.48	12.38	-16.56
Q5	19.19	34.79	4.39

Multivariate AFT model adjusted age, sex, race, TDI, physical activity, fasting time, diet score, drinking status, lowering lipids drugs, antihypertensives, insulin, and diabetes.

[a] Additional adjustment of body mass index in the model.

Table S5. Adjusted median time difference for SCA occurrence in the second, third, fourth, and highest quintile groups compared to the TyG index and TyG-BMI lowest quintile (Q1) groups according to sex.

Categories	Median Difference (months)	95 % Lower CI	95 % Upper CI	P for trend
		**Men**		
		**TyG[a]**		**0.304**
Q2	5.65	23.73	-11.16	
Q3	4.03	21.57	-12.3	
Q4	3.58	20.81	-12.47	
Q5	9.83	27.07	-6.26	
		**TyG-BMI**		**0.08**
Q2	-2.78	11.98	-16.48	
Q3	1.69	15.97	-11.63	
Q4	-2.57	11.38	-15.57	
Q5	10.82	25.67	-3.04	
		**Women[b]**		
		**TyG[a]**		**0.006**
Q2	24.81	61.43	-8.54	
Q3	18.41	55.23	-15.06	
Q4	36.77	75.09	1.91	
Q5	51.02	91.65	14.14	
		**TyG-BMI**		**0.05**
Q2	-11.45	16.39	-36.89	
Q3	-1.95	26.46	-27.94	
Q4	-6.79	21.61	-32.74	
Q5	23.44	51.99	-2.84	

Multivariate AFT model adjusted age, sex, race, TDI, physical activity, fasting time, diet score, drinking status, lowering lipids drugs, antihypertensives, insulin, and diabetes.

[a] Indicates additional adjustments for body mass index.

[b] Indicates additional adjustments for menopausal status.

Table S6. Multivariate COX regression analyses of TyG index and TyG-BMI with risk of SCA.

	**Including coronary artery disease [a]**		**Excluding participants within two years of follow-up**	
TyG [b]	HR (95% CI)	P-value	HR (95% CI)	P-value
Q1	Reference		Reference	
Q2	1.14 (0.97-1.35)	0.107	1.15 (0.96-1.38)	0.137
Q3	1.10 (0.94-1.30)	0.234	1.12 (0.93-1.34)	0.234
Q4	1.16 (0.99-1.36)	0.068	1.18 (0.98-1.41)	0.079
Q5	1.22 (1.04-1.43)	0.014	1.28 (1.07-1.53)	0.007
P for trend	0.025		0.01	
Per SD increase	1.07 (1.02-1.12)	0.004	1.09 (1.04-1.15)	0.001
TyG-BMI				
Q1	Reference		Reference	
Q2	0.96 (0.82-1.14)	0.660	0.95 (0.79-1.14)	0.563
Q3	1.01 (0.86-1.18)	0.932	1.04 (0.87-1.24)	0.676
Q4	0.97 (0.83-1.14)	0.725	0.97 (0.82-1.16)	0.743
Q5	1.19 (1.02-1.39)	0.028	1.25 (1.06-1.48)	0.01
P for trend	0.014		0.004	
Per SD increase	1.11 (1.06-1.16)	<0.001	1.14 (1.09-1.20)	<0.001

Multivariate COX regression analyses adjusted for age, sex, race, TDI, physical activity, fasting time, diet score, drinking status, lowering lipids drugs, antihypertensives, insulin, and diabetes.

[a] Indicates additional adjustments for coronary artery disease.

[b] Indicates additional adjustments for body mass index.

Table S7. Multivariate Cox regression of TyG index and TyG-BMI and SCA risk after multiple imputations of five data sets.

TyG [a]	Data set 1	Data set 2	Data set 3	Data set 4	Data set 5	Pooled results	P value
	HR (95% CI)	HR (95% CI)	HR (95% CI)	HR (95% CI)	HR (95% CI)	HR (95% CI)	P-value
Q1	Ref.	Ref.	Ref.	Ref.	Ref.	Ref.	
Q2	1.16 (0.96-1.4)	1.16 (0.97-1.4)	1.16 (0.97-1.4)	1.16 (0.97-1.4)	1.16 (0.96-1.39)	1.16 (0.97-1.39)	0.114
Q3	1.12 (0.94-1.35)	1.12 (0.93-1.34)	1.12 (0.93-1.34)	1.13 (0.94-1.35)	1.12 (0.93-1.34)	1.12 (0.94-1.35)	0.214
Q4	1.18 (0.98-1.41)	1.17 (0.98-1.4)	1.17 (0.98-1.4)	1.18 (0.99-1.41)	1.17 (0.98-1.40)	1.17 (0.98-1.40)	0.80
Q5	1.31 (1.1-1.56)	1.30 (1.09-1.55)	1.30 (1.09-1.56)	1.32 (1.11-1.58)	1.30 (1.09-1.55)	1.30 (1.09-1.56)	0.003
P for trend	0.006	0.007	0.006	0.004	0.008		
Per SD increase	1.10 (1.04-1.15)	1.09 (1.04-1.15)	1.10 (1.04-1.15)	1.10 (1.05-1.16)	1.09 (1.04-1.15)	1.09 (1.04-1.15)	<0.001
TyG-BMI							
Q1	Ref.	Ref.	Ref.	Ref.	Ref.	Ref.	
Q2	0.95 (0.79-1.14)	0.95 (0.79-1.14)	0.95 (0.79-1.13)	0.95 (0.79-1.13)	0.95 (0.79-1.14)	0.95 (0.79-1.14)	0.579
Q3	1.06 (0.89-1.26)	1.06 (0.89-1.26)	1.05 (0.88-1.25)	1.05 (0.89-1.25)	1.08 (0.91-1.28)	1.06 (0.89-1.26)	0.513
Q4	0.97 (0.82-1.16)	0.98 (0.83-1.17)	0.97 (0.81-1.15)	0.98 (0.83-1.17)	1.0 (0.84-1.19)	0.98 (0.82-1.17)	0.843
Q5	1.26 (1.07-1.5)	1.27 (1.08-1.51)	1.26 (1.07-1.5)	1.30 (1.1-1.53)	1.29 (1.09-1.52)	1.28 (1.08-1.52)	0.004
P for trend	0.003	0.002	0.003	<0.001	0.001	<0.001	
Per SD increase	1.14 (1.08-1.19)	1.14 (1.09-1.2)	1.14 (1.08-1.2)	1.15 (1.10-1.21)	1.14 (1.09-1.2)	1.14 (1.09-1.2)	<0.001

Multivariate COX regression analyses adjusted for age, sex, race, TDI, physical activity, fasting time, diet score, diabetes, drinking status, lowering lipids drugs, insulin, and antihypertensive medication.

[a] Indicates additional adjustments for body mass index.

Table S8. Multivariate Cox regression models stratified by sex to investigate the association of TyG and TyG-BMI levels with the incidence of SCA

	**Men**		**Women** [a]	
TyG [b]	HR (95% CI)	P-value	HR (95% CI)	P-value
Q1				
Q2	1.08 (0.85-1.37)	0.52	1.25 (0.93-1.68)	0.146
Q3	1.06 (0.84-1.33)	0.642	1.18 (0.87-1.61)	0.282
Q4	1.05 (0.84-1.32)	0.675	1.39 (1.02-1.87)	0.035
Q5	1.14 (0.92-1.43)	0.236	1.56 (1.15-2.12)	0.005
P for trend	0.304		0.005	
Per SD increase	1.05 (0.99-1.12)	0.096	1.17 (1.07-1.28)	<0.001
TyG-BMI				
Q1				
Q2	0.95 (0.75-1.21)	0.705	0.89 (0.67-1.18)	0.419
Q3	1.03 (0.82-1.29)	0.806	0.98 (0.74-1.31)	0.912
Q4	0.96 (0.77-1.20)	0.716	0.94 (0.70-1.25)	0.662
Q5	1.19 (0.95-1.49)	0.128	1.28 (0.98-1.66)	0.069
P for trend	0.078		0.048	
Per SD increase	1.12 (1.05-1.19)	<0.001	1.15 (1.06-1.24)	<0.001

Multivariate Cox regression analyses adjusted for age, sex, race, TDI, physical activity, fasting time, diet score, diabetes, drinking status, lowering lipids drugs, insulin, and antihypertensives.

[a] Indicates additional adjustments for menopausal status.

[b] Indicates additional adjustments for body mass index.

Table S9. Multivariate Cox regression model to study the relationship between TyG levels and SCA incidence.

TyG	HR (95% CI)	P-value
Q1	Reference	
Q2	1.13 (0.94-1.36)	0.187
Q3	1.09 (0.90-1.31)	0.374
Q4	1.13 (0.94-1.36)	0.181
Q5	1.22 (1.02-1.46)	0.03
P for trend	0.048	
Per SD increase	1.07 (1.02-1.13)	0.007

Multivariate COX regression analyses adjusted for age, sex, race, TDI, waist circumference, physical activity, fasting time, diet score, drinking status, lowering lipids drugs, antihypertensives, insulin, and diabetes.

Figure S1. Directed acyclic graph of the link between TyG index and TyG-BMI and the onset of SCA.

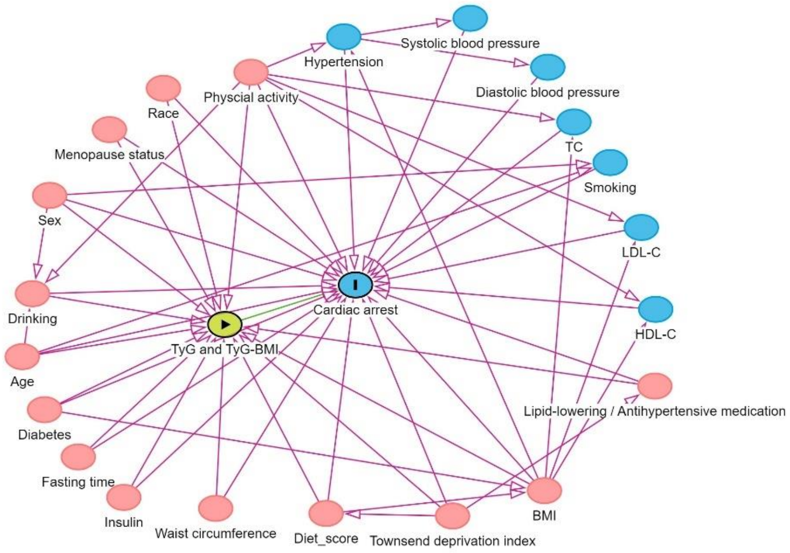

Diagram created with the help of DAGitty.net (www.dagitty.net). Minimal adjustment set: sex, age, race, BMI, waist circumference, TDI, fasting time, physical activity, diet score, diabetes, lowering lipids, antihypertensives, insulin, and drinking status.

Pink represents exposure factors of interest and blue represents outcomes of interest and potential mediators of exposure factor-outcome associations that should not be adjusted for in the main analyses.

Figure S2. Restricted cubic splines of TyG index, TyG-BMI and risk of SCA in women and men.

The results have been adjusted for age, sex, race, TDI, physical activity, fasting time, diet score, drinking status, lowering lipids drugs, antihypertensives, insulin, and diabetes. Additional adjustment for BMI in the relationship between TyG and SCA.

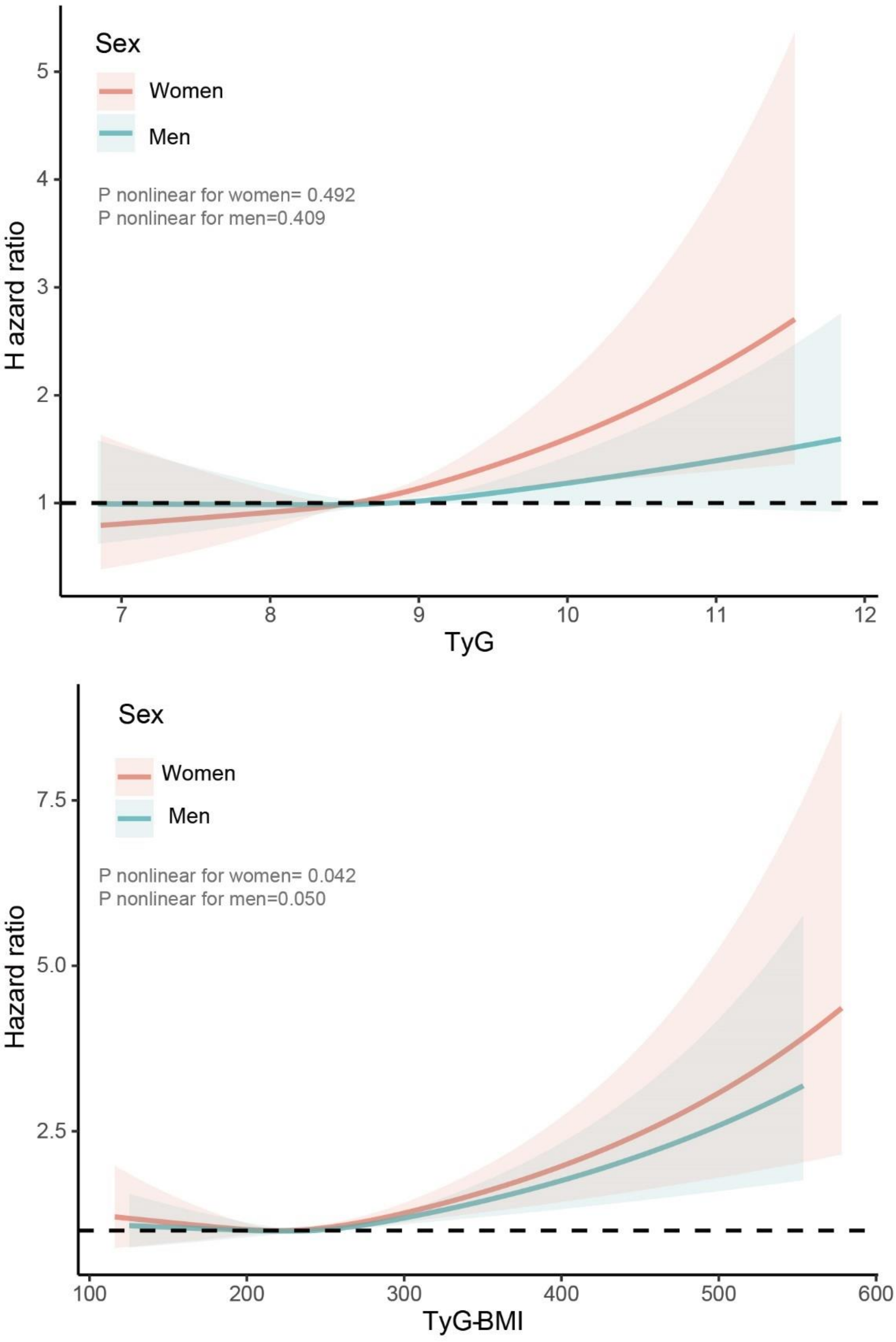

Supplement: Supplementary file 1 — Additional file1 (PDF 465 kb) [file 12933_2024_2275_MOESM1_ESM.pdf]
